# Variable Genome Sequences of the Murine Pneumotropic Virus (*Polyomaviridae*) Regulatory Region Isolated from an Infected Mouse Tissue Viral Suspension

**DOI:** 10.1128/genomeA.00405-16

**Published:** 2016-05-26

**Authors:** Jane E. Libbey, Robert S. Fujinami

**Affiliations:** Department of Pathology, University of Utah School of Medicine, Salt Lake City, Utah, USA

## Abstract

The murine pneumotropic virus genome, isolated from an infected murine tissue homogenate, was sequenced to completion. The lungs, liver, spleen, and kidneys were the source of the tissue homogenate in order to mirror the heterogeneity of the virus population *in vivo*. The regulatory region sequence was found to be highly variable.

## GENOME ANNOUNCEMENT

Murine pneumotropic virus (MPtV), which naturally infects mice (*Mus musculus*), was originally isolated and described by Kilham and Murphy (1, 2). Infection of newborn mice results in severe interstitial pneumonia, whereas infection of immunocompetent mice is clinically inapparent and is followed by lifelong persistence (3, 4). Sequencing of the MPtV genome isolated from infected mice has been performed by others (Mayer and Dörries [5], GenBank accession no. NC_001505; Ben Salem et al. [6], GenBank accession numbers KT987216 to KT987218), and sequencing of the pKV(37-1) clone encoding the MPtV genome has been performed by us (7) (GenBank accession no. EF186666). The regulatory region of MPtV, which contains the origin of viral DNA replication as well as all transcriptional regulatory sequences, was also sequenced previously (Zhang and Magnusson [8], GenBank accession numbers AJ517507 to AJ517510) to examine the heterogeneous nature of the enhancer segment.

We sequenced the entire MPtV genome obtained from John E. Greenlee (University of Utah) as a 10% suspension of infected mouse tissue in Hanks balanced salt solution (3). The MPtV genome was amplified by PCR, using *Pfu* DNA polymerase (Stratagene), from the infected-tissue suspension. Primers were designed to amplify the entire MPtV genome in three overlapping pieces. Two of the PCR products were directly sequenced without cloning. The third piece, containing the regulatory region, produced two PCR products and was therefore cloned by reamplifying the PCR product with primers containing the restriction enzyme sites for BamHI and SalI, digestion of the PCR product and pBluescript II SK(+/-) vector (Stratagene) with BamHI and SalI (Invitrogen), ligation, and transformation. Plasmid was prepared via the QIAprep spin miniprep kit (Qiagen). All sequencing was done by the Sequencing Facility at the University of Utah, Salt Lake City, UT.

The protein-coding region of the MPtV genome was identical to the pKV(37-1) plasmid (accession no. EF186666). The regulatory region of the MPtV genome was found to contain multiple sequences. We sequenced 17 clones and, in addition to sequences previously submitted to GenBank (accession no. AJ517507, one clone; accession no. AJ517509, four clones, one containing an additional G170A; accession no. AJ517510, one clone containing an additional deletion at positions 81 to 85 [*dl*81-85]; accession no. AJ517509 plus G272T of AJ517508, two clones), we found five sequences not in GenBank. One sequence had an insertion of the cellular transposable element (type B, *in*B) at nucleotide 142 with *dl*143-148, in conjunction with a duplication at positions 149 to 172 (*dp*149-172) (accession no. EF186668; four clones, one containing an additional G55T). A second sequence had the first 4 bp of the cellular transposable element [*in*A/B(1–4)] inserted at nucleotide 142, with the *dl*143-148 and the G272T transversion (accession no. EF186667; two clones, one containing an additional C122T mutation). A third sequence had *dl*143-163 (accession no. EF186670; one clone). The above-mentioned three sequences were previously described (8). Two additional sequences have not been described. One had a C122T mutation and a *dl*139-208 (accession no. EF186669; one clone), and the other had an insertion at nucleotide 141 of six nucleotides (GTTTAT), followed by a *dp*86-141 and accompanied by a *dl*142-182 (accession no. EF186671; one clone). This high variability in the regulatory region has been suggested to affect viral pathogenesis (8).

### Nucleotide sequence accession numbers.

The sequences of the various regulatory regions of MPtV isolated from an infected murine tissue homogenate have been deposited in GenBank under accession numbers EF186667 to EF186671. The sequence of the protein-coding region was identical to the plasmid sequence (Libbey and Fujinami [7], accession no. EF186666).
